# Adressing Energy Demand and Climate Change through the Second Law of Thermodynamics and LCA towards a Rational Use of Energy in Brazilian Households

**DOI:** 10.3390/e24111524

**Published:** 2022-10-25

**Authors:** Marina Torelli Reis Martins Pereira, Monica Carvalho, Carlos Eduardo Keutenedjian Mady

**Affiliations:** 1 School of Mechanical Engineering, University of Campinas, Campinas 13083-970, SP, Brazil; 2 Department of Renewable Energy Engineering, Federal University of Paraíba, João Pessoa 58051-900, PB, Brazil; 3 Department of Mechanical Engineering, Centro Universitário FEI, São Bernardo do Campo 09850-901, SP, Brazil

**Keywords:** thermodynamics, exergy analysis, life cycle assessment, energy, climate change, SDG 12

## Abstract

This study focuses on a typical Brazilian household through the lens of sustainable development, regarding energy demand and GHG emissions. The analysis encompasses both the direct and indirect energy, exergy consumption, and GHG emissions (quantified by life cycle assessment) associated with the usual routine of a household. The household is modeled as a thermodynamic system to evaluate inputs (food, electricity, fuels for transportation) and outputs (solid and liquid residues). The hypothesis is that each input and output contains CO${}_{2,eq}$ emissions and exergy derived from its physical-chemical characteristics or production chains. Each household appliance is modeled and tested as a function of external parameters. The contribution of several industries was obtained to the total GHG emissions and exergy flows entering and exiting the household (e.g., fuels for transportation, food, gas, electricity, wastewater treatment, solid waste). It was verified that urban transportation was the flow with the highest GHG and exergy intensity, ranging between 1.49 and 7.53 kgCO${}_{2,eq}$/day and achieving 94.7 MJ/day, almost five times higher than the calculated exergy demand due to electricity. The second largest flow in GHG emissions was food due to the characteristics of the production chains, ranging from 1.6 to 4.75 kgCO${}_{2,eq}$/day, depending on the adopted diet. On the other hand, the electricity presented low GHG emissions due to the main energy sources used to generate electricity, only 0.52 kgCO${}_{2,eq}$/day. Moreover, the chemical exergy of the solid waste was 9.7 MJ/day, and is not irrelevant compared to the other flows, representing an interesting improvement opportunity as it is entirely wasted in the baseline scenario.

## 1. Introduction

Adequate and rational energy use is the next challenge of society within the next years, as future generations should be able to access these resources with sufficient quality and minimal environmental impacts. In 2020, the Brazilian GHG emissions per capita associated with the energy mix were 1.87 ton CO${}_{2,eq}$/hab, with an annual growth of 1.4% [1]. These emissions are conditioned by the share of renewable sources in Brazil’s electricity mix, especially hydro.

In 2020, the share of renewable energy sources in electricity generation was 84.8% in Brazil, whereas its share in the domestic energy supply (energy matrix) was 48.4%. The share of non-renewable energy sources in electricity generation reflects climate conditions (rainfall regime) and energy demand, as most dispatchable thermal power plants are used as a backup in moments of less rainfall.

Despite increasing investments in renewable sources such as solar and wind, the share of renewable energy sources in the energy mix 2020 was 48.4%, not much higher than in 2011 (43.6%) [2]-the lowest mark was reached in 2014 due to droughts: 39.5%.

In 2020, the Brazilian residential sector was responsible for 10.8% of the final energy consumption of the country. The main energy source is electricity, followed by firewood-however, natural gas presented the highest increase in consumption compared to 2019 [3]. Regarding the overall energy consumption of the residential sector, the contribution of renewables is 67% [1].

It is essential to invest in energy efficiency and conservation aiming to decrease environmental burdens due to energy generation and use. In Brazil, the main initiatives targeting the consumers consist in the programs led by INMETRO-PROCEL and CONPET [4]. Among the actions implemented by these programs, an energy efficiency label (National Energy Conservation Label) deserves especial attention, aimed at certifying products and appliances and making the energy efficiency rates available to the public. A more detailed analysis of this programs may be found in [5].

In the field of energy efficiency, several authors highlight exergy analysis as an effective way to pinpoint losses [6] and quantify their magnitudes, besides being an essential guideline for policy-making [7] to achieve optimized resource utilization [8]. The rational use of natural resources by communities is based on the principles of thermodynamics and electrification [9].

Regarding the applications of exergy analysis, Mosquim et al. [10] quantified the destruction of exergy and exergy efficiency of the state of São Paulo (Southeast Brazil) as 28.3% and 12%, respectively, for the residential sector. These authors also verified that the exergy efficiency of the transportation sector since the 1970s was never over 10% [11]. Mosquim et al. [12] verified the effect of performance and efficiency tradeoffs, and the availability to obtain a more efficient sector when policy makers revise some restrictions or make accurate encouragements.

Chowdhury et al. [13] evaluated the exergy efficiency of the rural residential sector in Bangladesh, and the results ranged between 4.86% and 8.42%. However there are no details on the appliances used in the energy conversion processes. Jalil-Vega et al. [14] presented an optimization model for energy systems and services, and conducted a case study in the state of São Paulo with carbon restriction constraints. They achieved several scenarios that indicate a necessity for an increase of ethanol power vehicles; electric vehicles, both when the restriction is carbon emissions. Mady et al. [5] considered consumption patterns and appliances to quantify the exergy patterns of standard Brazilian households, concluding that the Second Law of Thermodynamics with the concept of extended exergy is a complementary indicator when comparing different technologies to obtain the same effect.

The Life Cycle Assessment (LCA) methodology has been frequently used to complement thermodynamic assessments, and is responsible for mapping the potential environmental impacts associated with a process, product or activity. With LCA, it is possible to quantify the impacts of consumption habits in several contexts, such as transportation [15], residue management [16,17] and food production systems [18,19,20], etc. Going a step further, the concept of “extended exergy analysis” [21] combines exergy and LCA in a single analysis.

The objective of the study presented herein is to combine exergy and LCA to study different characteristics of a household. The distinguishing feature of this study is to quantify GHG emissions (CO${}_{2,eq}$) and exergy efficiency for the Brazilian case, concerning consumption habits and production patterns. Quantitative data are used to establish a baseline scenario, followed by the identification of improvement opportunities afterwards. A quantitative study is carried out for a baseline scenario in which goods and services are a part of a typical Brazilian household (including its consumption rate). A sensitivity analysis is conducted using exergy efficiency on the final electricity use by typical appliances, aiming to compare the exergy behavior.

## 2. Material and Methods

The exergy analysis combines the first and second laws of thermodynamics and is given by Equation (1) for a control volume in a simplified form, disregarding kinetic and potential energy variations. Environmental conditions are denoted by $T_{0}$, $p_{0}$, and $\mu_{0}$,

(1) $$\frac{dB_{CV}}{dt}=\sum {\left(\dot{m}b\right)}_{inlet}-\sum {\left(\dot{m}b\right)}_{out}+\sum_{i}{\dot{Q}}_{i}\left(1-\frac{T_{0}}{T_{i}}\right)-{\dot{W}}_{CV}-{\dot{B}}_{dest}$$

$B_{CV}$ is the exergy of a control volume in kJ, $\dot{m}b$ refers to the exergy flows carried in or out of the control volume by a mass flow rate, ${\dot{Q}}_{i}$ is the heat transfer rate for surface *i* at a temperature *T*${}_{i}$, ${\dot{W}}_{CV}$ is the power and ${\dot{B}}_{dest}$ is the destroyed exergy of the process. As stated in Oliveira Junior [22], exergy quantifies the difference between a body or stream compared to the surroundings [23]. Exergy can include physical (temperature and pressure differences) and chemical (composition differences) shares, and is not conserved (as a matter of fact, it is always degraded by irreversibilities). Exergy efficiency therefore represents the ratio of useful work performed compared to the total exergy input as indicated by Equation (2).

(2) $$\eta_{ex}=\frac{\dot{B_{useful}}}{\dot{B_{input}}}$$

Exergy efficiency quantifies the efficiency of a process considering a reference state and the degradation of the quality of energy (exergy degradation).

**Figure 1:**
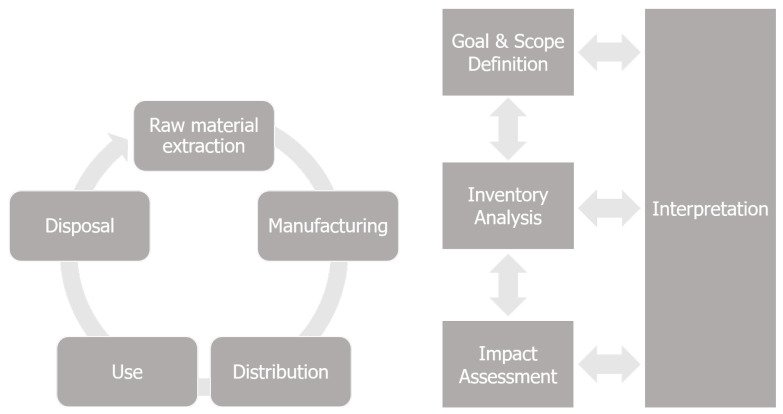
Life-cycle assessment framework.

Regarding LCA, it is employed to quantify the potential environmental impacts associated with a product, process, or activity. LCA can encompass the entire life cycle, from the extraction of raw materials to final disposal, and is standardized by ISO 14040 [24] and ISO 14044 [25]. Figure 1 shows a scheme of LCA’s framework, in which the left side depicts the stages that can be part of the analysis, and the right side shows the steps included in an LCA.

LCA is divided into four steps [26]: definition of goal and scope, analysis of inventory, impact assessment, and interpretation. In the first step, the purpose of the LCA is defined, along with its boundaries. The functional unit, to which all inputs and outputs relate to, is also defined. The second step accounts for the material and energy flows associated with the functional unit, and the third step selects an environmental impact assessment method to express the environmental impacts. Finally, results are obtained and interpreted, and conclusions are drawn.

**Figure 2:**
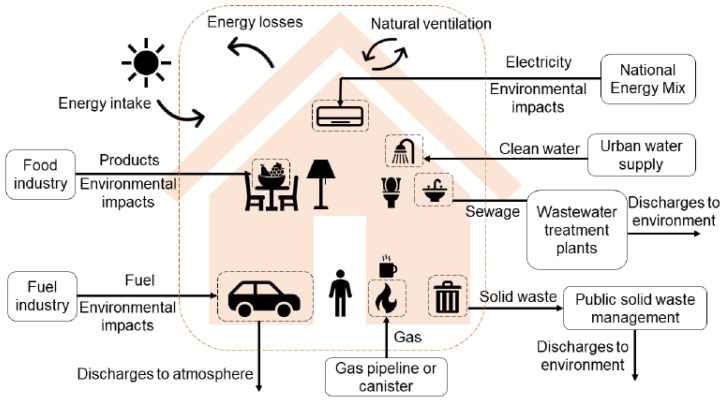
Proposed control volume with its inputs and outputs, and respective suppliers.

Herein a control volume is traced surrounding a theoretical typical Brazilian household, accounting for inputs and outputs that are necessary to fulfil everyday needs. For each input and output, embedded exergy (in MJ) and GHG emissions (in kgCO${}_{2,eq}$) are assigned. For food, the embedded exergy represents the accumulated energy used during the production process. For fuels, electricity, and waste, the embedded exergy is the actual exergy of the flow (combination of physical and chemical exergy shares). The proposed control volume is illustrated in Figure 2.

A sensitivity analysis is conducted with the Engineering Equation Solver [27] to evaluate the exergy efficiency of the everyday energy conversion processes within the control volume. The exergy efficiency of the household was calculated as a function of the efficiency of these processes, considering the contribution of each appliance to the overall consumption, following [28].

### 2.1. Residential Appliances

This subsection refers to the consumption of electricity for the air conditioner, electric shower, and refrigerator. The stove is evaluated as a non-electrical appliance used to heat water. Wall simulations used manufacturer data and information disclosed by INMETRO [29].

**Figure 3:**
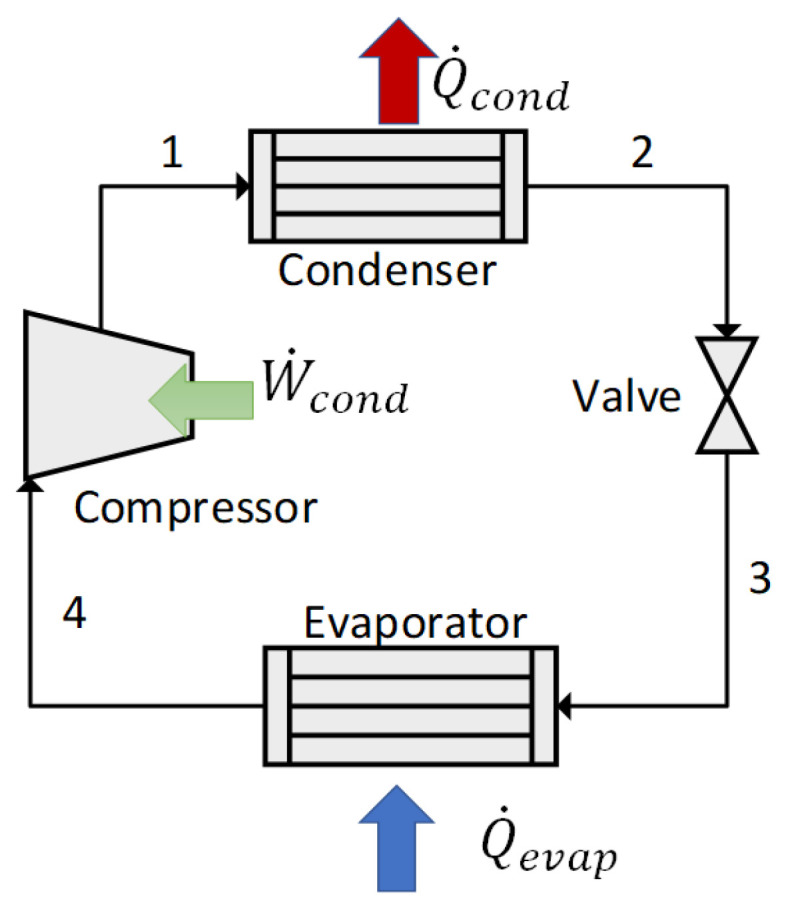
Vapor compression system, used in the air conditioner and refrigerator.

Aside from the consumption and efficiency information disclosed by manufacturers and INMETRO, the simulations were carried out considering specific regulations and ordinances published by the latter, concerning test methods [30,31]. Figure 3 indicates the vapor compression system used to represent the air conditioner and refrigerator.

Equations (3) and (4) are used to simulate the vapor compression systems within the air conditioner and the refrigerator. The term ${\dot{Q}}_{evap}$ refers to the heat removed from the environment, and ${\dot{W}}_{comp}$ to the power consumed by the compressor. In Equation (4) $T_{0}$ is the reference temperature for the exergy analysis (actual ambient temperature) and $T_{evap}$ is the evaporator temperature.

(3) $$COP=\frac{{\dot{Q}}_{evap}}{{\dot{W}}_{comp}}$$

(4) $$\eta_{ex}=COP\left(1-\frac{T_{0}}{T_{ev}}\right)$$

The daily use is considered to be 8 h. For the air conditioner, it is assumed that the compressor operates during 75% of the time [32]. The comfort temperature considered is 23 °C, and no air conditioning is required for temperatures close to thermal comfort temperature. The evaporator inlet temperature is considered constant at 10 °C. The fluids used in the simulations for the refrigerator and the air conditioner are R-134a and R-410a, respectively. For the refrigerator, the evaporator unit is inside the freezer compartment, with a constant temperature of −6 °C.

**Figure 4:**
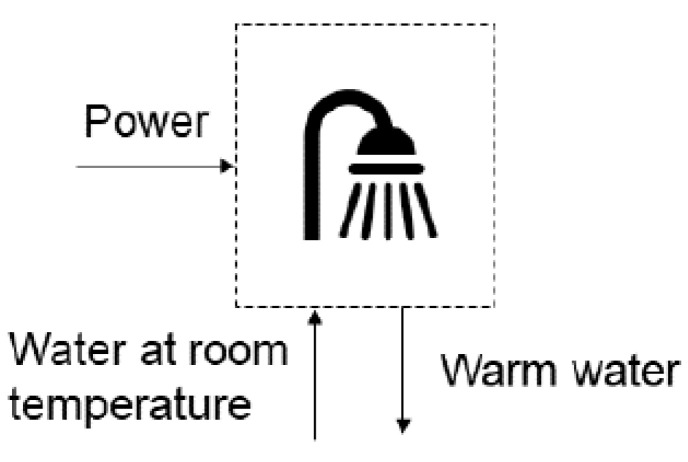
Electric shower, heating water by resistive heating.

For the electric shower (Figure 4), Equations (5) and (6) [5] are employed. The daily use is considered as 10 minutes, with water flow rate at 4 L/min and water temperature increase ranging from 10 °C to 30 °C, inversely proportional to ambient temperature.

(5) $$\eta_{en}=\frac{{\dot{m}}_{water}\Delta h_{water}}{{\dot{W}}_{electric}}$$

(6) $$\eta_{en}=\frac{{\dot{m}}_{water}\Delta b_{water}}{{\dot{W}}_{electric}}$$

**Figure 5:**
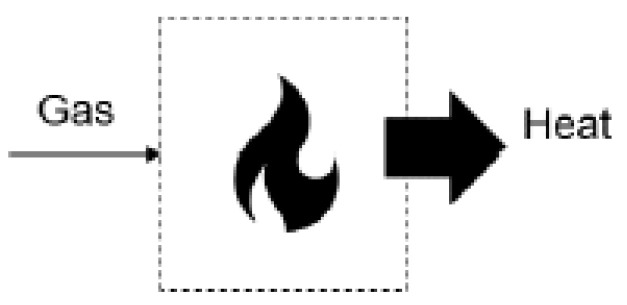
Representation of a stove and general water heaters that operate on natural gas.

In Equations (5) and (6), ${\dot{m}}_{water}\Delta b_{water}$ is the variation of physical exergy of water and $\Delta h_{water}$ is the variation of enthalpy of water. The term ${\dot{W}}_{electric}$ is the energy and exergy input. For the stove, Figure 5 depicts a simplification of the process in which chemical exergy of gas is converted into exergy associated with heat or an increase in the temperature of water.

Equation (7) is used for the stove. The simulation considers that 0.5 kg of water is heated from 25 °C until 100 °C. The usual energy efficiency is 63% for stove burners (data obtained from the Brazilian Labeling Program for grade-A stoves [29]

(7) $$\eta_{ex}=\frac{{\dot{m}}_{water}\Delta b_{water}}{{\dot{m}}_{NG}b_{NG}}$$

${\dot{m}}_{NG}b_{NG}$ is the chemical exergy of natural gas and ${\dot{m}}_{water}\Delta b_{water}$ is the increase in the exergy of water.

### 2.2. Supply Chains of Food, Electricity and Fuel

**Figure 6:**
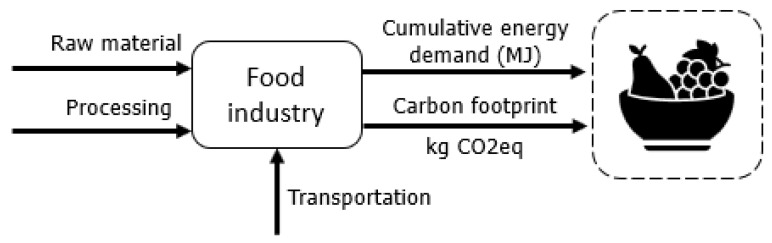
Simplified relationship between agri-food businesses and the supplied products.

**Figure 7:**
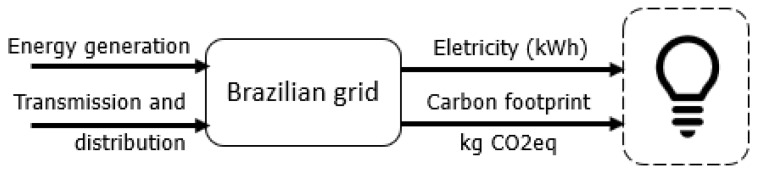
Simplified relationship between the national electric grid and the electricity supplied to consumers.

**Figure 8:**
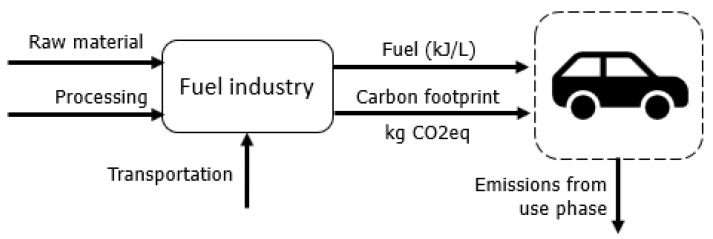
Simplified relationship between the demanded fuels for transportation and its respective industry.

**Figure 9:**
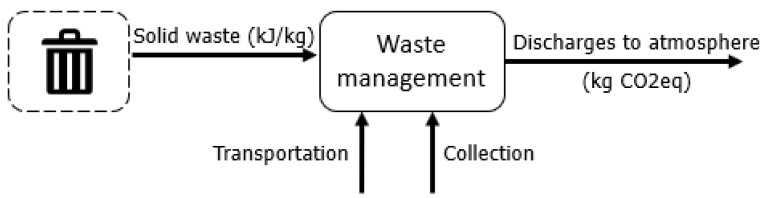
Simplified relationship between the solid residues produced and the management services.

The supply chains of electricity, food, fuel, and waste are illustrated in Figure 6, Figure 7, Figure 8 and Figure 9 in a summarized way.

Due to the variability of results for the environmental impacts of agri-food systems, two types of diet are compared: animal protein-based (referred to as standard) and a vegetarian diet, following [33]. Different food groups and corresponding amounts within the food pyramids are considered, following [34], and [35] for both types of diet.

The production chains of agri-food systems comprise clearing an area to cultivate food or grow animals, and providing all the raw materials and infrastructure for operation (e.g., machinery and buildings). As the production system operates, there are other demands: feed for the animals, fertilizers, materials for packaging, plus utilities such as water for irrigation and electricity and fuels (renewable and non-renewable) for machinery operation. When the product is ready for the market, it is transported to distribution centers and purchased by consumers. The product that reaches consumers has a history of processes with embedded energy and GHG emissions.

These processes are highly variable among the several types of food considered, and can have different rates and processes of land-use change, different needs for water, electricity and transportation, with different technologies employed in irrigation, packaging, processing, etc. Different energy conversion processes are required to achieve the final product, including industrial scale production, family farming, or other forms farming. Authors such as Gokbulak et al. [36] conducted evaluations of possible gains such as 1 t of fast food with a correct drying could generate approximately 3.5 GW. Moreover, Nazir et al. [37] established possible reductions in the food chain production to decrease the CO${}_{2,eq}$ of the bovine meat production.

Validated LCA studies have been adopted to represent the real production chains of common types of food consumed in Brazil (considering standard and vegetarian diets): for sugarcane and its products (e.g., sugar) 0.23 kgCO${}_{2,eq}$/kg [38,39], rice 1.14 kgCO${}_{2,eq}$/kg [19,40], beans 0.31 kgCO${}_{2,eq}$/kg [41], soybean 10.58 kgCO${}_{2,eq}$/kg [18,42,43], banana 0.42 kgCO${}_{2,eq}$/kg [44], urban and rural fresh vegetables [45], carrots [46], pasta 0.46 kgCO${}_{2,eq}$/kg [47], milk [48], lettuce 0.22 kgCO${}_{2,eq}$/kg [20] olive oil in Greece 1.2 kgCO${}_{2,eq}$/kg [49], and bread 0.02 kgCO${}_{2,eq}$/kg [50]. The effects of deforestation in the Brazilian scenario followed [51], and a comparison of organic and conventional farming systems followed [52].

Concerning Brazilian food habits, Carvalho et al. [53] remarked that the population of São Paulo state consumes excessive amounts of meat (above the recommended 500 g per week). Monteiro et al. [54] mentioned that only one in eight interviewees reported eating the recommended daily amounts of vegetables and fruits in Brazil. Philippi et al. [34] and Venti-Johnston [35] proposed food pyramids, which are employed to model food consumption herein.

The primary source of energy of the residential sector is electricity (used for general appliances and lighting), followed by wood (mainly for cooking). Wood has been progressively substituted by liquefied petroleum gas and natural gas for cooking and sometimes water heating [3].

Regarding electricity generation and supply, the Brazilian electricity mix is strongly dependent on hydro power, complemented by fossil fueled thermal power plants (mostly natural gas). The electricity mix considered for the analyses is 64.9% hydro-power, 9.3% natural gas, 3.3% coal, 2.5% nuclear, 8.4% biomass, 2% oil, 1% solar and 8.6% wind [1]. The GHG emissions associated with the 2019 Brazilian electricity mix were 104.1 kgCO${}_{2,eq}$/MWh [3].

Electricity is one of the most important chains of production [10]. It is important to highlight that the exergy content of electricity is equal to its energy. Electricity is demanded by the television, laptop, coffee machine, washing machine, refrigerator, electrical shower and air conditioner. However, only the air conditioner, shower and refrigerator will be studied with computational models in the sensitivity analysis. The average use of the remaining electrical appliances will follow manufacturer data and information from INMETRO [29]. The consumption habits are in accordance with PROCEL [55].

It is considered that 100% of the electricity demanded is supplied by the electric grid, and therefore there is no on-site electricity generation. As illustrated by Figure 7, the grid is an interconnected system that receives the electricity generated by each power plant, and redistributes it throughout the connected subsystems through transmission lines. Local distributors supply electricity to the consumers.

The fuels used for transportation are represented by their low heating value (LHV) and its adaptation for the exergy basis. A well-to-wheel approach is considered for the quantification of GHG emissions. The LHV and GHG emissions for each fuel are shown in Table 1.

Daily transportation patterns considered typical middle-class practice, with road transportation in a passenger (private) car [57] for the city of São Paulo.

### 2.3. Water and Waste Management

**Figure 10:**
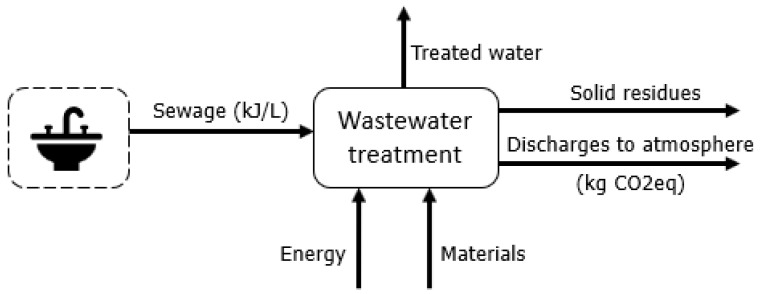
Simplified relationship between the liquid residues produced and the management services.

Regarding waste management of solids and liquids, the chemical exergy of the flows is considered, as this exergy is already a measurement of the changes caused in the environment by these outputs. Regarding water (Figure 10), it is assumed that 100% of the daily water demand (except for drinking) is simply discarded and then directed to wastewater treatment plants [58,59]. Solid waste is not sorted [60] (solid waste in Brazil does not usually have a proper destination [61]). The exergy assessment adopted data from [16,62]. For the GHG emissions, Refs. [17,63] were followed.

## 3. Results and Discussion

### 3.1. Greenhouse Gas Emissions

**Figure 11:**
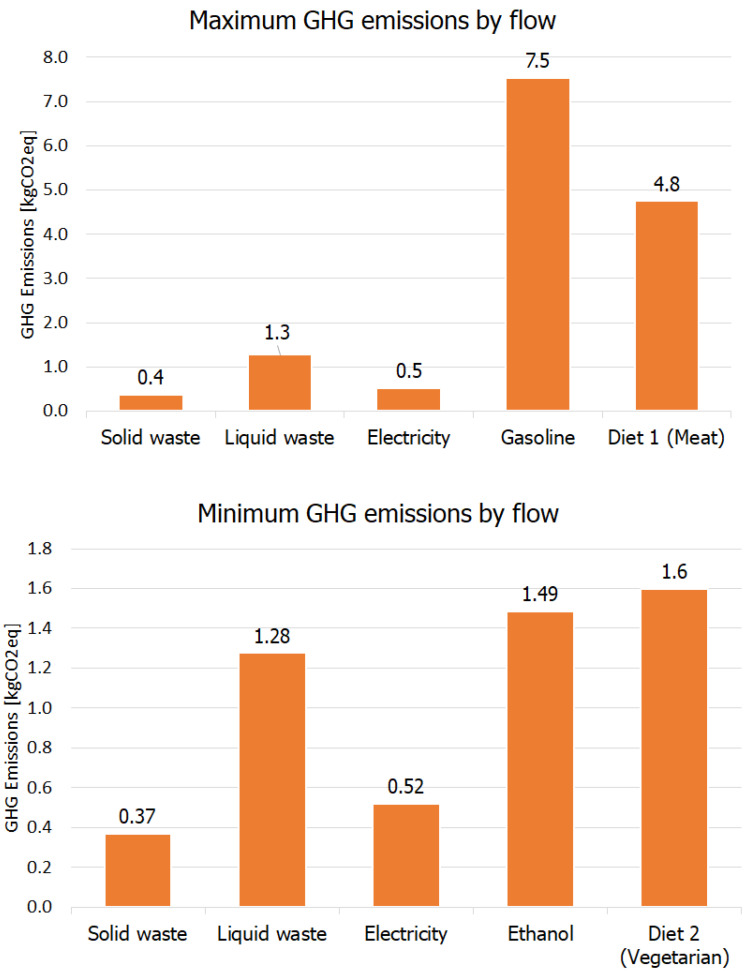
Maximum and minimum daily GHG emissions calculated, using two different scenarios for fuels and food consumption, in kgCO${}_{2,eq}$.

Considering the aforedescribed activities of daily life, Figure 11 shows the results of the GHG emissions associated with diet, consumption of electricity, fuels, and waste. Two situations are possible: maximum emissions (which considered the consumption of gasoline, and a standard diet), and minimum emissions (which considered the consumption of ethanol fuel, and a vegetarian diet).

From Figure 11 it is observed that on an annual basis, the GHG emissions can be 5209 kg CO${}_{2,eq}$/year for the maximum emission scenario (realistic), or as low as 1824 kg CO${}_{2,eq}$/year considering a vegetarian diet and use of ethanol fuel.

It is recognized that public transportation entails lower environmental impacts, but for convenience purposes people decide to invest in a car due to the quality of transportation and time of each trip [12].

The transportation sector is still one of the most significant source of emissions in Brazil. Figure 11 shows that ethanol can help mitigate the issue, and is an adequate intermediate solution for Brazil. Electric cars are still a tiny percentage of the Brazilian fleet [12] due to high prices. More research is still required to establish the impact of electric vehicles on the decarbonization process of the Brazilian society, as fossil fuels are employed part of the year to generate electricity.

The GHG emissions of ethanol correspond to only 20% of gasoline emissions [12]. Mady et al. [5] has pointed out that the National Energy Conservation Label presents ethanol with zero emissions (probably only considering operation-related emissions). Either way, the replacement of gasoline with ethanol is already capable of contributing significantly to decarbonization, especially considering a generalization of incentives and mass adoption of biofuels by the population.

Dietary habits also have a significant impact on GHG emissions.The GHG emissions of the vegetarian diet herein considered corresponded to 34% of the GHG emissions of the standard diet. Although a personal habit and preference, it encompasses an entire production chain, with significant impacts associated with the origin of the protein consumed [64,65,66]. Global food systems can be responsible for 30% of total anthropogenic GHG emissions, and this can be mitigated by shifting towards a plant-based diet and eating locally [67].

Individual changes in dietary habits can contribute to mitigate climate change, and semi-vegetarian and light semi-vegetarian diets have emerged due to animal-rights and ecological concerns [68] is desirable, especially if such changes are generalized. Meatless Mondays is a campaign that aims to decrease meat consumption by 15%, with relevant results obtained: for example, in a large urban US school district, entrees served during meatless Mondays were associated with −74% GHG emissions [69].

It must be mentioned that the intention is not to impose a specific diet, but only to test and investigate each example. Even though changes in dietary habits are highly attractive from an environmental viewpoint, eating habits are a very complex matter because they are shaped by a variety of factors: personal taste (flavor, smell, texture, general appearance of a dish), culture (customs and religion), lifestyle (availability to prepare food, convenience, education, family habits), and other factors such as public policies, price and availability of different foods [70].

However, a vegetarian-based diet is not automatically environmentally-friendly: Reis and Mady [71] verified that the cultivation of soy can impact land use, as new areas are cleared (deforestation). It is essential to provide such information to the consumer, as already occurs in the European Union [72]. This is an opportunity for the consumer to achieve better understanding and context about the sustainability debate, and make more informed choices, when possible [73].

Regarding the consumption of electricity, this parameter is favored by the high percentage of hydro and other renewables in the mix. The emissions associated with the consumption of 1 MWh of electricity from the Brazilian electric grid are much lower than for China (698.6 kgCO${}_{2,eq}$/MWh), the USA (386.9 kgCO${}_{2,eq}$/MWh), and the European Union (285.0 kgCO${}_{2,eq}$/MWh).

For waste disposal, the high emissions associated with solid waste are due to poor management practices, with limited recycling and reuse in society. However, regarding liquid waste, São Paulo is the state with the highest percentage of proper sewage treatment in Brazil. Emissions associated with waste disposal (solid and liquid) are lower compared to the other flows (except electricity). The emissions associated with liquid waste disposal are higher than for solid residues [61] due to the production of methane. Methane is an energy resource that, when not harnessed for energy purposes, is entirely wasted. Therefore, it is not only possible, but advantageous, that waste disposal is integrated to the country’s energy matrix, contributing to extract the maximum thermodynamic potential of all energy resources and diversify the energy mix.

Other practices can contribute to enhance the environmental performance (reduce GHG emissions) of waste disposal, such as biological treatment, digestion, composting, and recycling. Ref. [17] simulated several alternatives for the solid waste management sector and found that the best scenario in terms of GHG emissions involved increased recycling rates and digestion with the capture of methane for energy generation. Digestion and gas capture can also be employed in sewage treatment plants.

### 3.2. Overall Exergy Analysis

**Figure 12:**
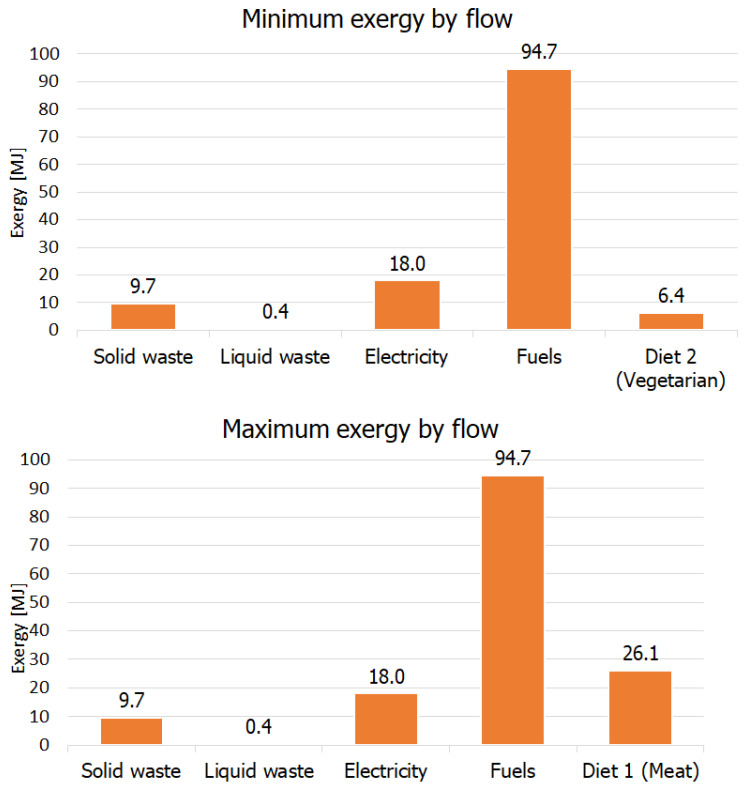
Minimum and maximum exergy calculated for each flow, using two different scenarios for fuels and food consumption.

The exergy flows are shown in Figure 12, on a daily and individual basis. When compared to Figure 11, there is an evident change in the contribution of fuels due to their exergy intensity. The exergy consumed in vehicles presents higher capability to perform work than the residues of the house. Solid and liquid residues can and should undergo treatments to increase exergy intensity.

The minimum, maximum, and average exergy values for a household are presented in Table 2.

Analysis of Table 2 indicates that there is not an intense gap among scenarios (the difference is only due to the diet option). Although different fuels were adopted in the scenarios studied, there is no change in the total exergy demanded for transportation. The analysis considers similar efficiency or mass (ethanol presents a lower energy intensity, compensated by higher consumption in km/L) [12]. For electricity and waste disposal the values remain the same.

In the Brazilian household model considered, individual transportation presented the highest energy and exergy intensities. As aforementioned, if public transportation was considered, these values are likely to decrease, as the demand is shared by all the passengers. Public transportation has already been evaluated as a strategy to conserve energy and reduce emissions in Canada [74]

From an exergy point of view, adopting policies to decrease the general interest by individual transportation is advantageous to reduce the demand for energy resources, leading to lower energy intensity and efficiency in the country. Moreover, when considering the GHG emission results by fuel, a hotspot was identified in this study, susceptible to improvement by a wide range of different practices and public policies to achieve a more efficient and sustainable society.

### 3.3. Sensitivity Analysis of Electricity Consumption by the Household Appliances

This subsection focuses on specific domestic appliances, which composed the exergy efficiency of the household. The electricity consumption of the typical household encompassed the use of electric shower, television, laptop, microwave, coffee machine, washing machine, air conditioner and refrigerator. The total consumption was approximately 150 kWh/month.

These results were compared to the residential sector’s electricity consumption data for 2022, as disclosed by EPE [75]. The results obtained herein were expected to be under average data [75], as lighting was not considered in the model and neither were other electrical appliances.

Table 3 shows the average data obtained herein, data from [75], and the difference.

The average obtained herein was close to official data. As the typical Brazilian household modeled followed São Paulo behavior (Southeast), the highest similarity was reached when comparing with Southeast Brazil data. The average consumption was also compared to monthly data provided by the same source [75], and the differences are presented by Table 4.

The smallest observed difference was 9 kWh in July, followed by August and June, with 11 and 14 kWh, respectively. During these months, it is winter in Brazil. This comparison makes sense considering the tested temperature range, limited to 29 °C, since the historical temperature range is between 12.9 °C and 27.3 °C, during the winter and considering these three months. On the other hand, during the summer, the historical average temperatures surpass 30 °C in most areas of the country [76].

Regarding the simulations for each appliance, environmental temperatures were between 12 and 29 °C. By calculating the household’s average efficiency as a function of these appliances and considering the contribution of the appliances to overall consumption [28], the average exergy efficiency was calculated as 13%. This result shows that 87% of the total exergy that enters a middle-class house is destroyed by basic appliances. This demonstrates and highlights the need for policies to increase this efficiency and motivate the population to choose more efficient appliances (not solely based on the equipment, but considering the form of conversion).

**Figure 13:**
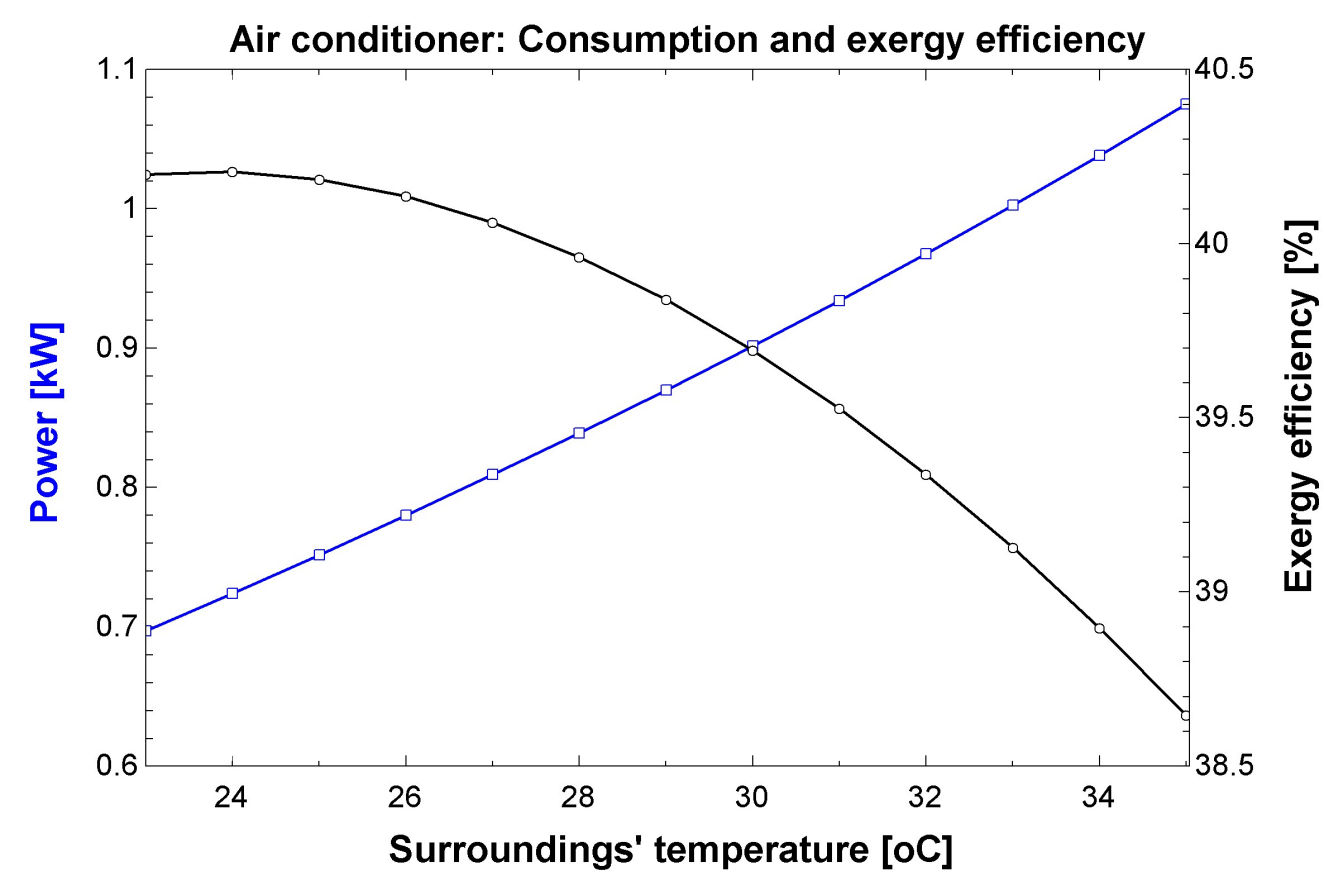
Results obtained in the simulation for the air conditioner.

For the air conditioner, its operation was restricted to temperatures over the thermal comfort temperature established (23 °C). The efficiency varied between 38 and 40%, whereas the power consumption range was 700 W–1100 W, for a thermal load between 2500 and 2600 W. Figure 13 shows the results of the air conditioner simulations. Official data disclosed by INMETRO for an equivalent equipment employing R-410a report consumption of 760 W for a thermal load of 2637 W [29]. The computational model employed herein is compatible with the parameters of the real tests.

**Figure 14:**
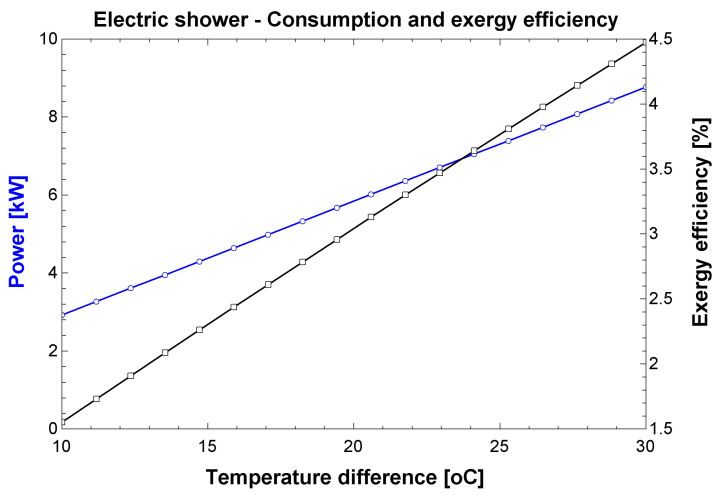
Simulation results for the shower.

The simulation results for the shower are presented in Figure 14. The simulation considered the increase in water temperature, considering that higher increases (up to 30 °C) will occur in colder locations and seasons. Less intense increases will occur in hotter locations and seasons. The electrical power of the shower varies between 3 and 8 kW, which is compatible to the range disclosed by INMETRO for electrical showers [29]. The average monthly consumption was 29 kWh considering the conditions mentioned in the Methods section. The exergy efficiency of the shower is calculated between 1.5% and 4.5%. Such low values are expected because the mechanism to obtain the useful effect is resistive heating, which transforms a highly noble input (electricity) into a more disorganized output (heat). From an exergy viewpoint, this technology is not a good mechanism to achieve the desired output.

Concerning the fridge (refrigerator), the obtained power ranges between 49 and 78 W for the simulated temperature range (12–29 °C). Considering a daily use of 8 h, the average monthly consumption is approximately 17 kWh. The power consumption increases in hotter days, as expected, while the exergy efficiency decreases (inversely proportional behavior).

**Figure 15:**
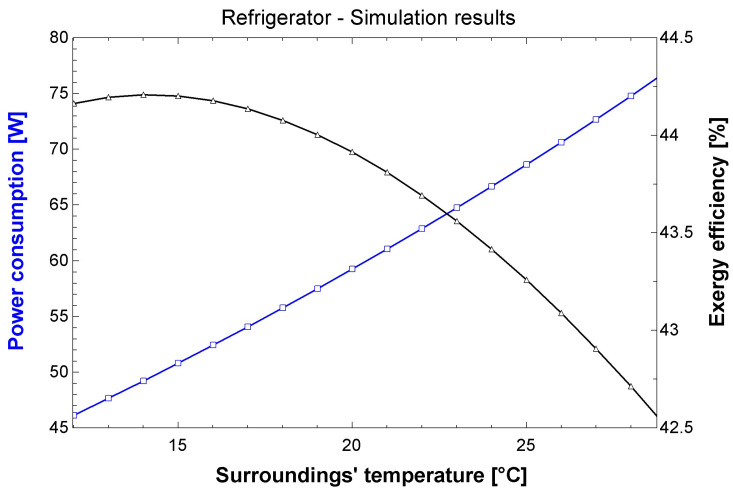
Simulation results for the refrigerator.

For comparison purposes, the manufacturer indicates a consumption of 23.9 kWh/month [29]. The results obtained herein (Figure 15) are slightly lower, however. Manufacturer data were obtained during tests at 32 °C [30], while the simulations in this study used a maximum temperature of 29 °C. The exergy behavior of the air conditioner is close to the refrigerator’s as power and efficiency are inversely proportional.

**Figure 16:**
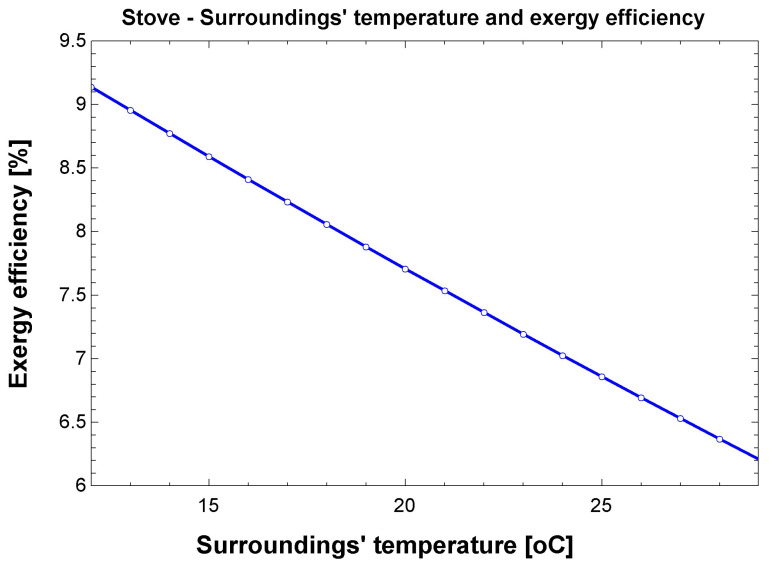
Results obtained in the simulation for the stove.

For the stove, the simulation results are depicted in Figure 16. The exergy efficiency range is 6–10%, decreasing as room temperature increases. Greater differences in the useful effect as compared to the environment state lead to higher efficiencies for this case as well as for the shower. The efficiency of the stove is higher than the shower’s, although both were tested for the same desired output (heating water). It is possible to conclude that, in terms of exergy, the combustion of a gas is a better technological choice than resistive heating.

It is possible to notice, from the results observed in this session, that plenty of opportunities are ahead towards a generalized improvement in the results. Moreover, this paper is linked to some of the Sustainable Development Goals.

Sustainable Development Goal 12 can be cited in this matter, as it aims for sustainable consumption and production patterns in general, and specially for its targets concerning sustainable resource management, access to information by the public, and waste reduction and management. Concerning the energy-related topics in this project, Sustainable Development Goal 7 (“clean and affordable energy”) is clearly linked, specially regarding energy efficiency and expanding the access to clean energy sources. Lastly, climate-related topics represented by Sustainable Development Goal 13 (“climate action”) are present throughout the paper’s scope and analysis, and the improvement in this matter is seen by the authors as a consequence achievable by the former two.

## 4. Conclusions

This study simulated a Brazilian household regarding GHG emissions and exergy in its everyday flows, as well as its overall exergy efficiency. The assessment encompassed the exergy behaviors of the appliances used in domestic energy conversion processes, and a 13% overall exergy efficiency was obtained as a function of these appliances. The results of the exergy analysis provided several insights unable to reach through simple energy analysis.

Regarding the flows analyzed, urban transportation presented the highest GHG and exergy intensities. Passenger cars (individual transportation) were considered to operate on gasoline and ethanol, to which the latter presented significantly lower GHG emissions. Public policies should be employed as strategies to improve and reduce the GHG and exergy intensities of the transportation sector as a whole. Besides carpooling and public transportation systems, suggestions can encompass non-motorized or alternative transportation. As demonstrated by the results, it is essential to motivate energy transition in this sector, replacing fossil fuels by biofuels or electricity. For the latter, the efficiency can be strongly improved with gains that are not achievable by the employment of internal combustion engine vehicles.

The second most relevant flow was associated with food, with two dietary options, in which protein was supplied by animals or vegetables. Recognizing that the most GHG-intensive product was beef, two diets were tested and the vegetarian diet presented lower GHG emissions. The authors acknowledge the complexity surrounding the feasibility of achieving such a change in habits in large scale, and recommend the application of policies to restrict land-use change as much as possible, specially in areas that are close to forest biomes.

Another interesting improvement opportunity identified was in waste management, given that for both solid and liquid waste, methane is generated and entirely wasted in baseline scenarios.

Electricity consumption was not GHG-intensive, due to the low-carbon electricity mix in Brazil. The authors recommend the continuity of regulations encouraging the adoption and dissemination of renewables, such as wind, solar and biomass, whose integration into the energy mix is rather recent when compared to traditional sources.

Finally, the need to optimize transportation stood out as an improvement opportunity. Flows that are typically ignored (waste) present interesting potentials for energy generation. Furthermore, the diet choice and its associated chain can heavily burden the products. This type of information should be available to consumers, to motivate informed decisions and to organize and demand better conditions from producers.

Future studies can focus on a interdisciplinary analysis that contemplates public policies aiming to achieve the desired improvements in the results and a deepening in the consumption drivers and markets responsible for meeting the consumers needs.

## Figures and Tables

**Table 1:** Lower Heating Value (LHV) and greenhouse gas emissions (GHG), from well-to-wheel for selected fuels. Data obtained in [56].

Fuel	LHV (MJ/L)	GHG Emissions (gCO${}_{2,eq}$/km)
Gasoline	28.9	159.83
Ethanol	21.3	31.53
Diesel	35.6	274.59

**Table 2:** Maximum, minimum and average exergy flows associated with a Brazilian household.

Exergy [MJ]	Daily	Yearly (365 Days)
Max	148.91	54,353
Min	129.22	47,166
Avg	139.07	50,760

**Table 3:** Comparison between official electricity consumption data provided by EPE (2021) and calculated results, for a Brazilian household, with data obtained from [75].

Zone	Average Official Consumption 2021 [kWh]	Estimated Consumption [kWh]	Difference [kWh]
North	183	150	33
Northeast	130	150	−21
Southeast	175	150	23
South	187	150	34
Central-west	154	150	39

**Table 4:** Monthly comparison between official electricity consumption data provided by EPE (2021) and calculated results, for a Brazilian household, with data obtained from [75].

Scenario	Jan	Feb	Mar	Apr	May	Jun	Jul	Aug	Sep	Oct	Nov	Dec
Official [kWh]	196	186	189	190	167	164	159	161	176	169	166	177
Difference [kWh]	46	36	39	40	17	14	9	11	26	19	16	27

## Data Availability

Not applicable.
